# Supplementary material for: Preoperative Assessment of Perianal Fistulas with Combined Magnetic Resonance and Tridimensional Endoanal Ultrasound: A Prospective Study
Source: Diagnostics (Basel). 2023 Sep 3;13(17):2851. doi: 10.3390/diagnostics13172851 (PMC10486944; doi:10.3390/diagnostics13172851)
Supplement: Supplementary file 1 [file diagnostics-13-02851-s001.zip › diagnostics-2571564-supplementary.pdf]

TABLE S1: Pelvic MRI Protocols for Perianal Fistula Evaluation

Sequences and Slice Orientation	No. of Signal Averages	FOV (mm)	Imaging Plane Direction	Acquired Matrix (Frequency × Phase)	Slice Thickness (mm)	TR/TE
Coronal single-shot turbo spin-echo (fast spin-echo) (localizer)	2	340 × 380	Right-left × Head-foot	316 × 247	5 mm 1.6gap	3000–5000/180
Axial high-resolution T2-weighted turbo spin-echo (fast spin-echo)	2	360 × 340	Right-left × anterior-posterior	800 × 759	4 mm 1gap	3000–5000/80
Sagittal T2-weighted turbo spin-echo (fast spin-echo) with fat suppression	3	200 × 200	Head-foot × anterior-posterior	400 × 233	6 mm 1.7gap	3000–5000/80
Axial T2-weighted turbo spin-echo (fast spin-echo) with fat suppression	4	200 × 160	Right-left × anterior-posterior	256 × 133	4 mm1 gap	3000–5000/80
Axial STIR (optional)	1	200 × 160	Right-left × anterior-posterior	256 × 149	4 mm1 gap	2500–6000/30
Sagittal STIR	3	200 × 200	Head-foot × anterior-posterior	400 × 233	6 mm 1.7gap	3000–5000/80
Axial T1-weighted turbo spin-echo (fast spin-echo)	2	200 × 160	Right-left × anterior-posterior	400 × 226	4 mm 1gap	500–800/11
Axial T1-weighted 3D gradient-recalled echo with fat suppression with and without IV contrast agent	4	200 × 160	Right-left × anterior-posterior	224 × 178	1.8	3.6/1.8
Sagittal 3D gradient-recalled echo with fat suppression	4	200 × 160	Foot-head × anterior-posterior	224 × 178	1.8	3.6/1.8
